# Energy consumption modeling of industrial robot based on simulated power data and parameter identification

**DOI:** 10.1177/1687814018773852

**Published:** 2018-05-04

**Authors:** Aiming Liu, Huan Liu, Bitao Yao, Wenjun Xu, Ming Yang

**Affiliations:** 1 School of Information Engineering, Wuhan University of Technology, Wuhan, China; 2 Hubei Key Laboratory of Broadband Wireless Communication and Sensor Networks, Wuhan University of Technology, Wuhan, China; 3 School of Mechanical and Electronic Engineering, Wuhan University of Technology, Wuhan, China; 4 Faculty of Engineering, Environment and Computing, Coventry University, Coventry, UK

**Keywords:** Energy consumption, industrial robot, parameter identification, power

## Abstract

Industrial robots consume a considerable amount of energy in the manufacturing industry. As sustainable manufacturing is one of the directions of manufacturing, the energy efficiency of the industrial robots should be considered. The energy consumption model is the key to analyze and improve the energy efficiency of industrial robots. This article focuses on the energy consumption characteristic analysis and energy consumption modeling of the industrial robot. The inertial and friction parameters which are the base of energy consumption modeling need to be identified. Since the torque which is usually used for parameter identification may be unavailable, we propose to use the software-simulated power data to perform the parameter identification and model the energy consumption of the robot. Then, comprehensive simulations are carried out to verify the effectiveness of the proposed method for parameter identification and the energy consumption model. The relation between the speed of the robot and its energy consumption is also analyzed.

## Introduction

Sustainable development has been attracting much attention since the United Nations published the Bruntland^1^ report *Our Common Future* in 1987. Energy-efficient manufacturing is an important part of sustainable development. Manufacturing industry is considered as the world’s pillar industry and it consumes a large amount of energy.^2^ In the manufacturing industry, industrial robots are playing a more and more important role and consuming a significant amount of energy. Taking automotive sector for example, the electrical energy consumed by robots in the production phase of vehicles is about 8% of total energy consumption in the vehicles’ lifecycle in average.^3^ Therefore, it is meaningful to analyze the energy consumption characteristics and model the energy consumption of industrial robots to facilitate energy efficiency optimization.

Recently, many efforts have been made on the energy consumption analysis of industrial robot.^3–10^ Generally, there are various components of the robotic system that consume energy, such as the controller, the fans for cooling, the motor, and the friction of the robot joint. Mohammed et al.^5^ proposed to choose energy-efficient robot configuration to save the energy consumption of industrial robot. Rassolkin et al.^6^ and Brossog et al.^10^ analyzed the electricity consumption characteristics of industrial robots and compared their energy consumption in different working conditions, for example, tool weights, different trajectories, different workpiece positions, and different velocities. Meike et al.^3^ presented a detailed model of energy consumption of industrial robot, including the energy consumption of the converters, the DC bus, the inverters, the motors, and the electromechanical brakes. This model needs a large number of parameters, some of which are confidential and hard to obtain. Pellicciari et al.^8^ modeled the energy consumption of industrial robots and calculated the energy-optimal trajectories by means of constant time scaling, with the inertial parameters of the robot being already known. Brossog et al.^9^ used the Modelica-based simulation tool to create a digital model of a six-axis industrial robot and used experimental results to improve the model’s accuracy. The effect of robot operating parameters (i.e. payload and speed) on the energy consumption of the robot was also analyzed.

To model the energy consumption of the industrial robot, it is necessary to explore the robot’s dynamics. However, the inertial and friction parameters of industrial robots are confidential and not known to users. Therefore, it is needed to obtain these parameters by parameter identification, which attracts the attention of many researchers.^11–21^ Swevers et al.^18^ and Swevers and Samin^19^ proposed a method for parameter identification of robot based on periodic excitation. The joint velocity and acceleration is obtained based on a delicate method and is of high quality. The maximum likelihood estimation is used and simplified to weighted linear least squares estimation because of the high quality of the joint velocity and acceleration. The joint torque is usually needed in the parameter identification of the inertial and friction parameters of industrial robots. Paes et al.^20^ used the feedback motor current to identify the inertial and friction parameters of the industrial robot. The technical specifications such as motor torque constants, torque characteristics, transmission ratios, and efficiencies are needed to calculate the joint torques from the current measurements. Some of these technical specifications are unknown and neglected. Christoforou^21^ used sensor that measured the base reaction forces/torques to identify the inertial parameters of the robot online and to improve the trajectory tracking controllers.

Although many efforts have been put on the energy consumption modeling and parameter identification, there are some points that need to be explored further. First, the joint torque which is usually used in parameter identification cannot be obtained for some industrial robots, such as ABB robots. The power of the robot can be measured conveniently when there is power sensor (the wiring is simple because only the total power of the robot is needed using the method proposed in this article) or obtained from simulation software (such as RobotStudio developed by ABB). Due to the lack of power sensor, this article proposes a method to use the software-simulated power data of the robot as an alternative for parameter identification. The dynamic model and energy consumption model can then be obtained. Second, there lacks a study to test the effectiveness of the energy consumption model based on the dynamic model of the industrial robot. This article fills in this gap.

## Energy consumption characteristic analysis

### Energy flow of the industrial robot

**Figure 1.**
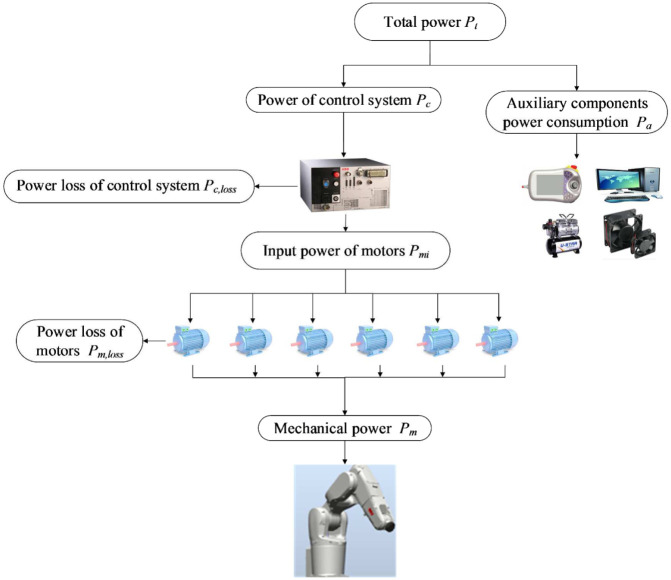
Energy flow of the industrial robot.

The industrial robot usually consumes AC power. The energy flow of an industrial robot is shown in Figure 1. The total power $P_{t}$ is consumed by the control system $P_{c}$ and the auxiliary components $P_{a}$ . The auxiliary components include the teach pendant, the air compressor for the pneumatic gripper, the fans for cooling the controller, and the PC which may be used to interact with the control system. The power of these auxiliary components is generally constant. The power supplied to the control system includes the power loss $P_{c,\mathrm{loss}}$ (the power consumption of the AC/DC converters, the DC bus, the inverters, etc.) and the power supplied to the motors of the robot $P_{\mathrm{mi}}$ . $P_{\mathrm{mi}}$ can be divided into two parts: one part is used to drive the motor $P_{m}$ and the other part is the power loss of the motors $P_{m,\mathrm{loss}}$ .

### Energy consumption of the motors

The motors nowadays used in industrial robots are generally permanent magnet synchronous motors and of high efficiency (between 83% and 92%).^22^ As shown by equation (1), the power losses include the copper loss, the iron loss, the mechanical losses, and the stray losses and can be expressed as

(1) $$P_{m,\mathrm{loss}}=P_{\mathrm{Fe}}+P_{\mathrm{copper}}+P_{F}+P_{\mathrm{stray}}$$

where $P_{m,\mathrm{loss}}$ is the power loss of the motor, $P_{\mathrm{Fe}}$ is the iron loss, $P_{\mathrm{copper}}$ is the copper loss, $P_{F}$ is the mechanical loss, and $P_{\mathrm{stray}}$ is the stray loss.

**Figure 2.**
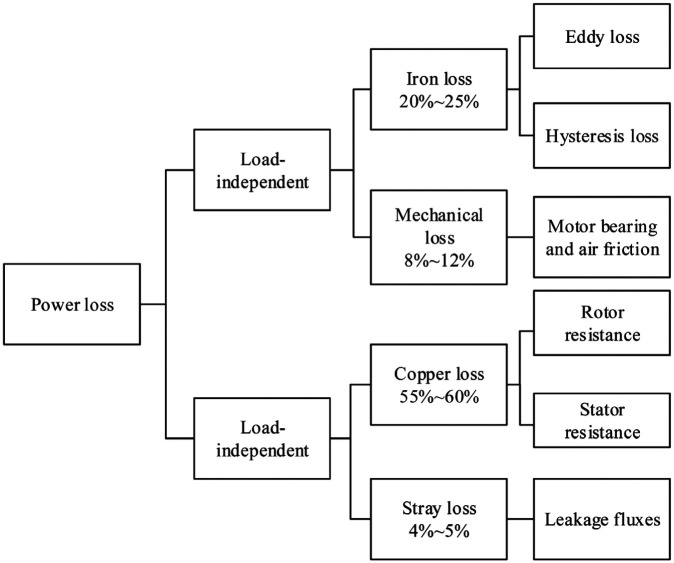
Power losses of the motor.^22^

As shown in Figure 2, the iron loss and the mechanical loss are nearly constant and independent of the load of the robot. They are determined by the materials, the manufacturing process of the motor, the input voltage, the structure of the motor, and so on. The iron loss is caused by the eddy current and the hysteresis in the stator of the motor. The mechanical losses are caused by the friction of the bearing in the motor and the air friction. The copper loss and the stray loss are dependent on the load of the robot. It is due to the resistance of the stator and the rotor. This power loss is transformed into heat. The stray losses are due to the leakage fluxes through the resistance of the stator winding.^22^

### Power driving the links

**Figure 3.**
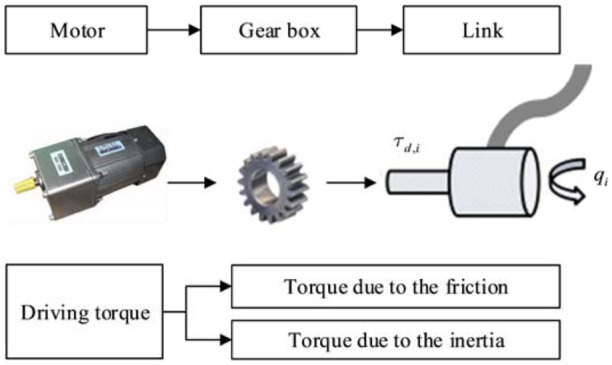
Drive system of the industrial robot.

The output power from the motor is used to drive the link of the robot. The diagram of the drive system is shown in Figure 3.

The torque $\tau_{d,i}$ driving the link *i* (*i* is the sequence number) is transmitted from the shaft of the motor, through the transmission system and to the link. $\tau_{d,i}$ can be expressed as

(2) $$\begin{array}{c} \tau_{d,i}=\tau_{\mathrm{dyn},i}+\tau_{f,i}\\ \tau_{\mathrm{dyn},i}={\left(D\left(q,\varphi \right)\right)}_{i}\overset{\cdot \cdot }{q}+{\left(C\left(q,\overset{\cdot }{q},\varphi \right)\right)}_{i}\overset{\cdot }{q}+{\left(G\left(q,\varphi \right)\right)}_{i}\\ \begin{array}{c} \tau_{f,i}=f_{c,i}\mathrm{sign}\left({\overset{\cdot }{q}}_{i}\right)+f_{v,i}{\overset{\cdot }{q}}_{i}\\ i=1,\ldots ,n \end{array} \end{array}$$

where $\tau_{\mathrm{dyn},i}$ is the torque due to the inertia of the robot, it is expressed in Newton–Euler equation, $\tau_{f,i}$ is the torque due to the friction of the joint i , $q\in R^{n}$ is the vector of joint positions, n is the degree of the robot, φ is the vector of inertial parameters, $D(q,\varphi )\in R^{n\times n}$ denotes the inertia matrix, $C(q,\overset{\cdot }{q},\varphi )\in R^{n\times n}$ is the Coriolis–centrifugal matrix, $G(q,\varphi )\in R^{n\times n}$ is the gravitational vector, $(\cdot {)}_{i}$ denotes the ith row of (·) , $\tau_{f,i}$ is the friction torque of the joint i , and $f_{c,i}$ and $f_{v,i}$ are the Coulomb and viscous friction parameters, respectively. This is a simple yet effective static friction model.

The power for driving the links can, therefore, be expressed as

(3) $$P_{d,i}=\tau_{d,i}{\overset{\cdot }{q}}_{i}=\tau_{\mathrm{dyn},i}{\overset{\cdot }{q}}_{i}+\tau_{f,i}{\overset{\cdot }{q}}_{i}$$

where $\tau_{\mathrm{dyn},i}{\overset{\cdot }{q}}_{i}$ and $\tau_{f,i}{\overset{\cdot }{q}}_{i}$ represent the power to overcome the inertia and the joint friction of the robot, respectively. When the joint is accelerating, the motor outputs power to the joint. When the joint is decelerating, the motor becomes a generator and the kinetic energy of the robot is transformed into electric energy. This energy can be restored by capacitors and recovered to accelerate other joints. However, for industrial robots the capacitance values of the capacitors are generally small and thus the energy recovery is limited. The generated electric energy is finally dissipated by the drain resistor in the servo system. In addition, a part of $P_{d,i}$ is transformed into power loss of the motor and heat due to the friction in the robot joint. Finally, all the power $P_{d,i}$ becomes heat.

This article focuses on the modeling of the energy consumption of the motors

(4) $$E=\int_{0}^{t}\sum_{i=1}^{n}\frac{P_{d,i}}{\mu_{i}}\mathrm{dt}$$

where $\mu_{i}$ is the efficiency of the motor and t is the time. This article models $P_{d,i}$ and then obtain the model (4).

## Energy consumption modeling

### Parameter identification based on power data

**Figure 4.**
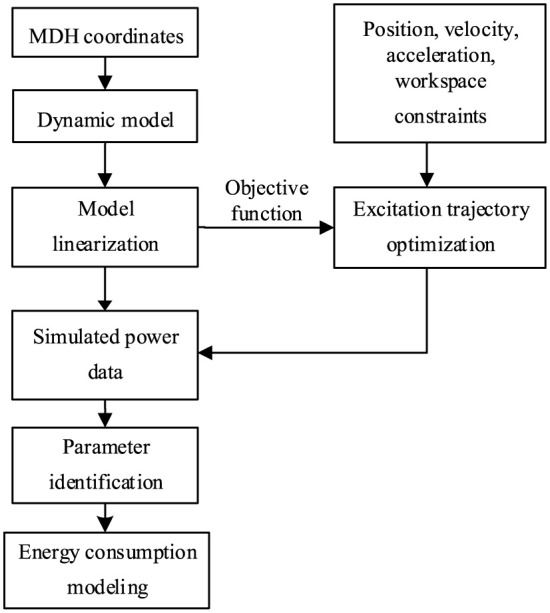
Steps for parameter identification.

To calculate **µ** in equation (4), it is necessary to know the inertial and friction parameters in equation (3). As the inertial and friction parameters of the industrial robot are unknown to users, parameter identification is needed. The joint torque of the robot is usually needed in the parameter identification. However, it may be unavailable for some robots. Therefore, a method of parameter identification based on simulated power data is proposed in this article. The flow diagram of parameter identification based on power data is shown in Figure 4.

### Modified Denavit–Hartenberg coordinates

**Figure 5.**
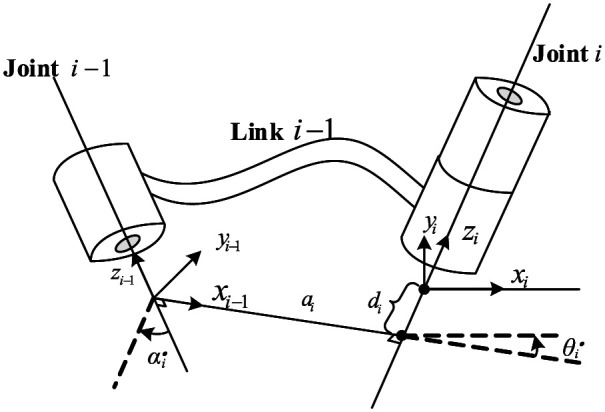
Modified D-H notation.

The notation of modified Denavit–Hartenberg (MDH) is shown in Figure 5, where i is the number of joints, $\alpha_{i}$ is the angle between $z_{i-1}$ and $z_{i}$ about $x_{i-1}$ , $a_{i}$ is the distance between $z_{i-1}$ and $z_{i}$ along $x_{i-1}$ , $\theta_{i}$ is the angle between $x_{i-1}$ and $x_{i}$ about $z_{i}$ , and $d_{i}$ is the distance between $x_{i-1}$ and $x_{i}$ along $z_{i}$ .

**Figure 6.**
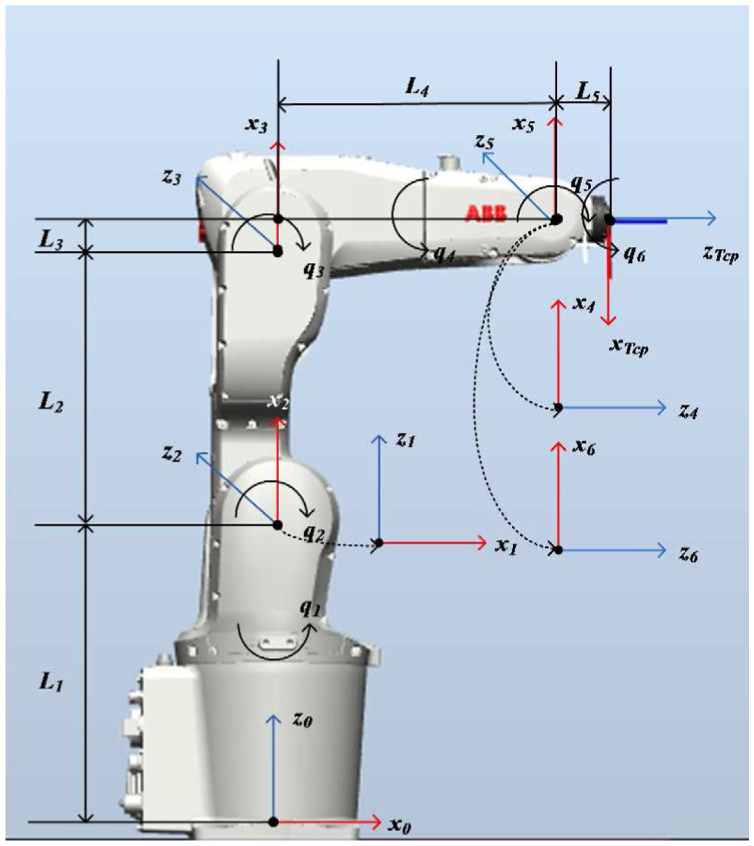
MDH coordinate frames of ABB IRB 1200.

This article uses ABB IRB 1200 to demonstrate the proposed method. The coordinates of the ABB IRB 1200 based on MDH method is shown in Figure 6. The MDH parameters of the robot are shown in Table 1.

**Table 1.** MDH parameters of ABB IRB 1200.

Link	$a_{i}\;(m)$	$\alpha_{i}\;(\mathrm{rad})$	$d_{i}\;(m)$	$\theta_{i}\;(\mathrm{rad})$
1	0	0	*L*_1_	0
2	0	–*π*/2	0	–*π*/2
3	*L*_2_	0	0	0
4	*L*_3_	–*π*/2	*L*_4_	0
5	0	*π*/2	0	0
6	0	–*π*/2	0	0
*TCP*	0	0	*L*_5_ *+* *h*	*π*

MDH: modified Denavit–Hartenberg.

### Linearization of dynamic model

Equation (2) can be linearized into equation (5) to facilitate the parameter identification^23^

(5) $$\tau_{d,i}={\left(W\left(q,\overset{\cdot }{q},\overset{\cdot \cdot }{q}\right)\right)}_{i}\Phi$$

where $W(q,\overset{\cdot }{q},\overset{\cdot \cdot }{q})$ is the observation matrix, $(W(q,\overset{\cdot }{q},\overset{\cdot \cdot }{q}){)}_{i}$ is the ith row of W , and Φ is the vector of inertial and friction parameters.

This article adopts least squares to identify the parameters in Φ . The estimated parameters can be expressed as

(6) $$\hat{\Phi }={\left(W^{T}W\right)}^{-1}W^{T}\tau_{d}+\gamma$$

where γ is the error vector of the parameter identification.

### Minimum set of inertial parameters

There are 10 inertial parameters $\left[\begin{array}{c} I_{i,\mathrm{xx}}\;I_{i,\mathrm{yy}}\;I_{i,\mathrm{zz}}\;I_{i,\mathrm{xy}}\;\begin{array}{c} I_{i,\mathrm{xz}}I_{i,\mathrm{yz}}\;mr_{\mathrm{ci},x}\;mr_{\mathrm{ci},y}\;mr_{\mathrm{ci},z}\;m_{i} \end{array} \end{array}\right]$ and two friction parameters $\left[\begin{array}{cc} f_{c,i} & f_{v,i} \end{array}\right]$ for each link of the robot, where $\left[\begin{array}{cccccc} I_{i,\mathrm{xx}} & I_{i,\mathrm{yy}} & I_{i,\mathrm{zz}} & I_{i,\mathrm{xy}} & I_{i,\mathrm{xz}} & I_{i,\mathrm{yz}} \end{array}\right]$ are the components of the inertia tensor of the link i and $\left[\begin{array}{ccc} mr_{\mathrm{ci},x} & mr_{\mathrm{ci},y} & mr_{\mathrm{ci},z} \end{array}\right]$ are the components of the first moment of the link i . Some inertial parameters have no effect on the dynamics of the robot and some inertial parameters can be regrouped into other inertial parameters. These parameters can be deleted. In order to reduce the number of inertial parameters and to improve the quality of parameter identification, it is necessary to calculate the minimum set of inertial parameters. The method for minimum set of inertial parameters can be calculated by Gautier and Khalil.^24^

### Excitation trajectory

Finite Fourier series are widely used as excitation trajectory for robotic parameter identification,^18^ as shown by

(7) $$\begin{array}{c} q_{i}(t)=q_{0,i}+\sum_{j=1}^{N}\left(a_{i,j}\sin\left(j2\pi \mathrm{ft}\right)+b_{i,j}\cos\left(j2\pi \mathrm{ft}\right)\right),\\ i=1,\ldots ,n \end{array}$$

where $q_{0,i}$ is the offset of the joint position, f is the base frequency of the trajectory, N is the number of items in the series, and $a_{i,j}$ and $b_{i,j}$ are the coefficients. The period of the excitation trajectory is T=1/f . The frequencies in the excitation trajectory need to be high enough to stimulate the inertial parameters of the robot. However, the frequencies should avoid the resonance frequencies of the robot structure.

The excitation trajectory can be optimized to improve the quality of parameter identification. The condition number of the observation matrix Φ is used as the criteria for excitation trajectory optimization. The smaller the condition number is, the higher the quality of parameter identification will be. The objective of excitation trajectory optimization is shown by equation (8). It subjects to the physical constraints of the robot, including the joint position constraint, the angular velocity constraint, the angular acceleration constraint, and the workspace constraint

(8) $$\begin{array}{c} \hat{\Psi }=\arg\;\min\;\mathrm{cond}\left(W\left(\Psi ,f\right)\right)\\ \mathrm{with}\left\{ \begin{array}{c} q_{\min}\leq q\left(T_{f},\Psi \right)\leq q_{\max}\\ {\overset{\cdot }{q}}_{\min}\leq \overset{\cdot }{q}\left(T_{f},\Psi \right)\leq {\overset{\cdot }{q}}_{\max}\\ {\overset{\cdot \cdot }{q}}_{\min}\leq \overset{\cdot \cdot }{q}\left(T_{f},\Psi \right)\leq {\overset{\cdot \cdot }{q}}_{\max}\\ \left\{s\left(q\left(T_{f},\Psi \right)\right)\right\}\subset S \end{array}\right. \end{array}$$

where Ψ is the vector of the optimal parameters in the excitation trajectory, S is the workspace of the robot, and s is the function calculating the forward kinematics. The MATLAB nonlinear optimization function *fmincon* can be used to optimize the excitation trajectory.

### Energy consumption modeling based on power data

As the torque may not be available for a portion of industrial robots, the power, which is easier to obtain, is a practical choice. The power of the motor i is shown as

(9) $$P_{m,i}=\frac{P_{d,i}}{\mu_{i}}=\frac{\tau_{d,i}}{\mu_{i}}{\overset{\cdot }{q}}_{i}$$

Combining equation (5) with equation (9), it can be obtained that

(10) $$P_{m,i}={\left(M\right)}_{i}\hat{\Phi }$$

where M is the new observation matrix, $(M{)}_{i}$ is the ith row of M , and M and $\hat{\Phi }$ can be calculated by

(11) $${\left(M\right)}_{i}={\left(W\left(q,\overset{\cdot }{q},\overset{\cdot \cdot }{q}\right)\right)}_{i}{\overset{\cdot }{q}}_{i}$$

(12) $$\hat{\Phi }=\frac{\Phi }{\mu }$$

where $\hat{\Phi }$ represents the new parameters to be identified and μ is the constant vector of the efficiencies of the motors.

Because $P_{d,i}$ can be hardly separated from $P_{m,i}$ , $P_{m,i}$ will be used in the parameter identification. Therefore, it is necessary to form the new parameters $\hat{\Phi }$ . The consequence is that $\hat{\Phi }$ is proportional to the real inertial and friction parameters of the robot and μ is absorbed by $\hat{\Phi }$ . Therefore, the calculation of the power of the motor is affected.

According to equations (10)–(12), the parameters of the robot can be identified as

(13) $$\hat{\Phi }\prime ={\left(M^{T}M\right)}^{-1}M^{T}P_{m}+\gamma \prime$$

where $P_{m}$ is the vector consisted by the power of each motor and γ′ is the error vector of parameter identification.

If the power of each motor can all be obtained separately, the unknown parameters of all the links of the robot can be identified by equation (13) at one time. If not, a step-by-step identification can be used, that is, identifying the parameters of the link n is the first step and then the parameters of the link i can be identified recursively based on the parameter identification results of the links i+1,…,n .

Based on equations (10) and (13), the energy consumption model of the motors can be expressed as

(14) $$\hat{E}(t)=\int_{0}^{t}\sum_{i=1}^{n}{\hat{P}}_{m,i}\mathrm{dt}=\int_{0}^{t}\sum_{i=1}^{n}{\left(M\right)}_{i}\hat{\Phi }\prime \mathrm{dt}$$

In manufacturing tasks, the tool and load of the robot may change according to the process in the tasks. In this article, the tool and load of the robot are considered as a part of link 6. Then, a new parameter identification will be performed. The process of parameter identification and energy consumption modeling are the same with the case where there is no tool or load.

## Simulation

### Simulation setup

**Figure 7.**
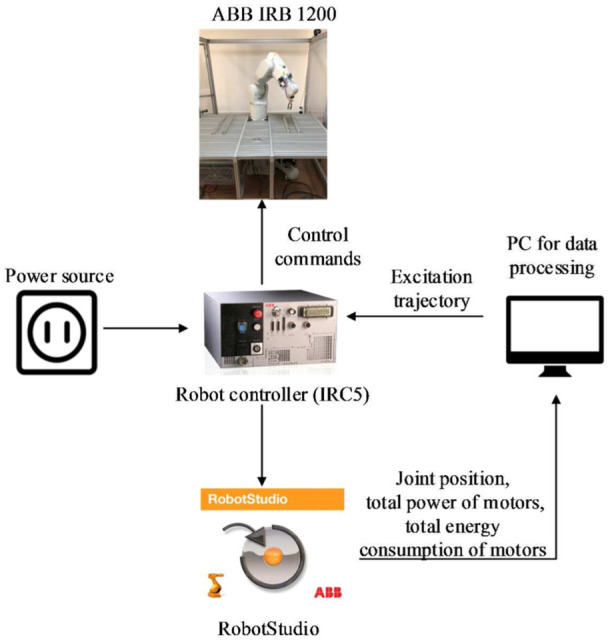
Simulation setup.

ABB IRB 1200 with 6 degrees of freedom (DOFs) is used to demonstrate parameter identification and energy consumption modeling based on power data. The power can be measured by power sensors or voltage and current sensors. As the simulation software RobotStudio provides the total power of all the motors, we use these data to perform parameter identification for convenience. The accuracy of the power data from RobotStudio affects the accuracy of parameter identification. Currently, we have not evaluated the errors of power data from RobotStudio, but this can be done in the future work. The joint position and the total energy consumption of the motor are also obtained from RobotStudio. The simulation setup is shown in Figure 7.

### Parameter identification and energy consumption modeling

This article uses step-by-step parameter identification. The parameter identification starts from the last link of the robot and backs to the first link. The results of the former steps are used in the later steps. Therefore, there will be six steps for parameter identification of ABB IRB 1200. At the step of parameter identification of the link i , the joints i+1,…,n are needed to be excited. In the simulation with loads, the loads are taken as a part of the last link.

**Figure 8.**
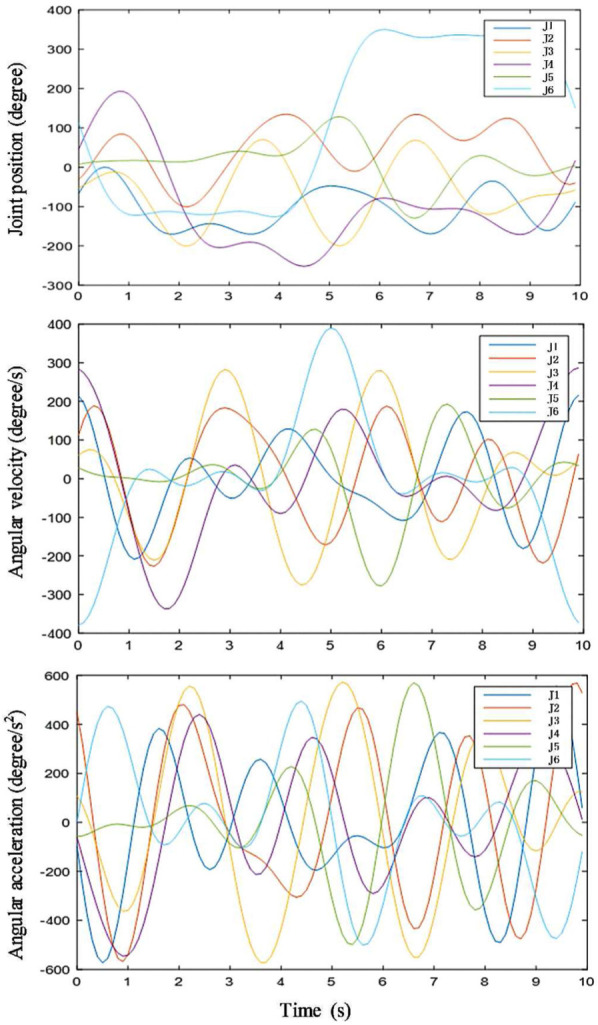
Optimized excitation trajectory.

**Figure 9.**
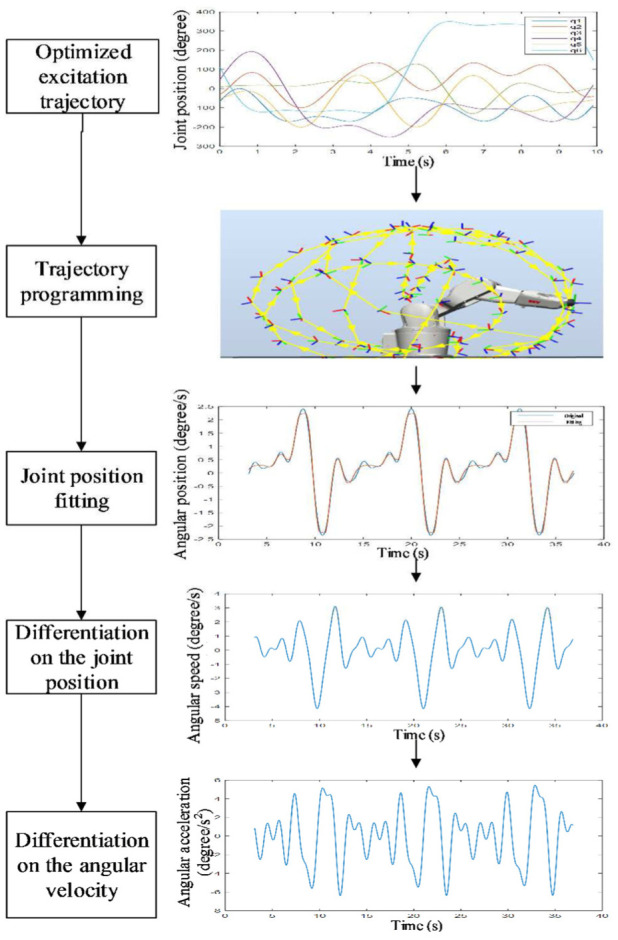
Signal processing in parameter identification.

For the excitation trajectory, the base frequency is chosen as 0.1 Hz. Five items in the Fourier series are used. An example of excitation trajectory in step 6 is shown in Figure 8. This is the optimized trajectory according to equation (8). As shown in Figure 9, the robot is commanded to move along the optimal trajectory. The total power of all the motors and the joint position are recorded to perform parameter identification based on equation (13). The data are sampled by 10 Hz. The recorded joint position is fitted by the Fourier series shown in equation (7) to reduce the noise in the data. Then the joint velocity and joint acceleration are calculated by differential operation on the joint position.

**Figure 10.**
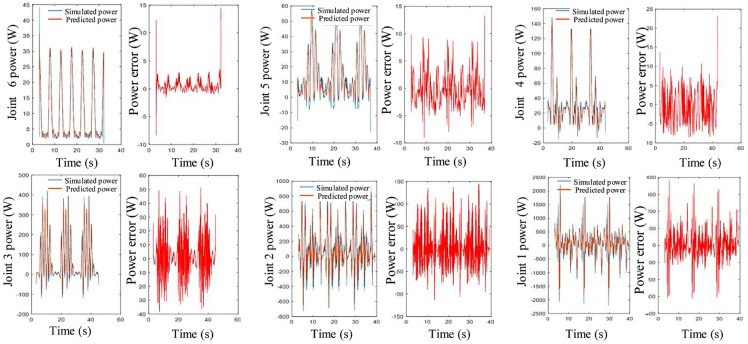
Simulated power and predicted power of each joint, and error of prediction.

The results of parameter identification of ABB IRB 1200 without load are shown in Table 2, where (·R)or(·r) mean the regrouped parameters when calculating the minimum set of inertial parameters. It should be pointed out that the results in Table 2 are not necessarily to be the physical values of the robot’s inertial or friction parameters. This is due to the constant μ in equation (12) which makes the estimated value proportional to the physical value of the inertial and friction parameters. Therefore, the results in Table 2 are not in SI unit and obtained when the data used in the parameter identification are in SI unit. The results in Table 2 are used to calculate the predicted power for each joint based on equation (10) and compared with the simulated power to verify the accuracy of the identified parameters. The predicted and simulated power of each link is shown in Figure 10.

**Table 2.** Results of parameter identification.

Link 6	$I_{6,\mathrm{xx}}R$	7.6123	Link 5	$I_{5,\mathrm{xx}}R$	4.4529
	$I_{6,\mathrm{zz}}R$	8.3414		$I_{5,\mathrm{zz}}R$	19.272
	$I_{6,\mathrm{xy}}R$	0.4194		$I_{5,\mathrm{xy}}R$	11.579
	$I_{6,\mathrm{xz}}$	−0.2057		$I_{5,\mathrm{xz}}$	−12.949
	$I_{6,\mathrm{yz}}R$	−2.9788		$I_{5,\mathrm{yz}}R$	6.8297
	$mr_{c6,x}$	−18.731		$mr_{c5,x}$	−21.598
	$mr_{c6,y}$	−22.716		$mr_{c5,y}$	−15.997
	$mr_{c6,z}$	−18.62		$f_{5,c}$	10.731
	$f_{6,c}$	4.8417		$f_{5,v}$	1.3441
	$f_{6,v}$	0.0855			
Link 4	$I_{4,\mathrm{xx}}R$	−11.686	Link 3	$I_{3,\mathrm{xx}}R$	−27.504
	$I_{4,\mathrm{zz}}R$	18.968		$I_{3,\mathrm{yy}}R$	−3.131
	$I_{4,\mathrm{xy}}R$	3.0248		$I_{3,\mathrm{zz}}$	4.8854
	$I_{4,\mathrm{xz}}$	2.912		$I_{3,\mathrm{xy}}R$	−8.1999
	$I_{4,\mathrm{yz}}R$	22.071		$mr_{c3,z}$	1.1607
	$mr_{c4,x}$	−2.2106		$f_{3,c}$	17.729
	$mr_{c4,y}$	−23.802		$f_{3,v}$	8.8749
	$mr_{c4,z}$	−47.591			
	$f_{4,c}$	19.512			
	$f_{4,v}$	1.0228			
Link 2	$I_{2,\mathrm{xx}}R$	34.674	Link 1	$I_{1,\mathrm{zz}}R$	24.853
	$I_{2,\mathrm{xy}}R$	10.437		$f_{1,c}$	94.423
	$I_{2,\mathrm{xz}}$	44.951		$f_{1,v}$	7.8637
	$I_{2,\mathrm{yz}}R$	30.549			
	$f_{2,c}$	42.063			
	$f_{2,v}$	74.855			

The results of parameter identification are used to calculate the predicted total energy consumption of the motors during the six steps of parameter identification based on equation (14). It is compared with the simulated energy consumption. The results are shown in Table 3. It shows that the error of energy consumption modeling increases from step 1 to step 6. This is because the error of parameter identification accumulates in the step-by-step method. However, the error is small and acceptable.

**Table 3.** The error of energy consumption modeling.

Step	Excited joints	Simulated total energy consumption (J)	Predicted total energy consumption (J)	Absolute error (J)	Relative error (%)
1	6	266.64	294.46	27.82	10.4
2	6, 5	751.07	706.28	40.79	5.4
3	6, 5, 4	1987.1	1936.9	50.2	2.5
4	6, 5, 4, 3	4978.3	5054	75.7	1.5
5	6, 5, 4, 3, 2	8509	8387.2	121.8	1.4
6	6, 5, 4, 3, 2, 1	12,486	12,317	231	1.8

**Figure 11.**
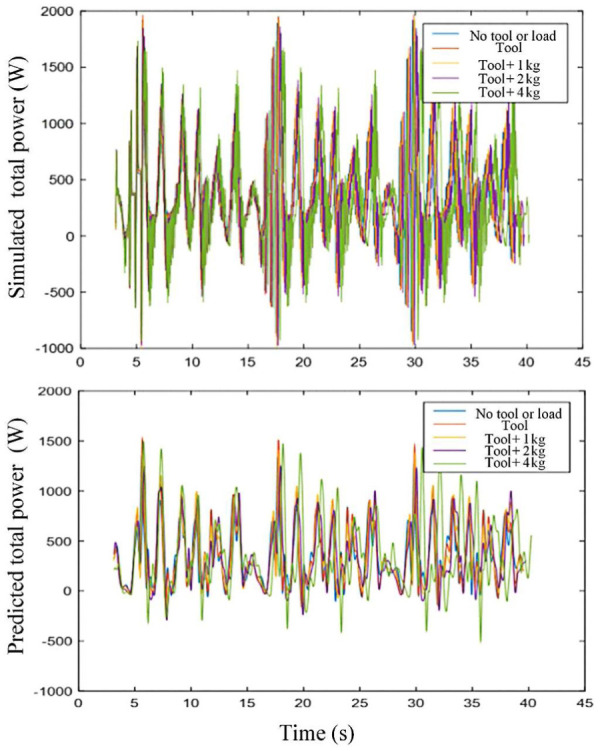
Simulated and predicted total power of the motors in different scenarios.

For scenarios where the robot carries tool and loads, the tool and loads are taken as a part of link 6. The power that is calculated based on parameter identification of the robot in five different scenarios (no tool or load, tool (1.32 kg), tool + 1-kg load, tool + 2-kg load, tool + 4-kg load, the trajectory is the same in all the scenarios) is shown in Figure 11. The energy consumption calculated based on equation (14) and the error of these scenarios are shown in Table 4. It is shown that the error in scenarios with tool and load is small enough. This verifies the accuracy of the energy consumption model.

**Table 4.** Modeling error of energy consumption in different scenarios.

Test	Simulated total energy consumption (J)	Predicted total energy consumption (J)	Absolute error (J)	Relative error (%)
No tool or load	12,486	12,517	31	0.25
Tool	12,591	12,706	115	0.91
Tool + 1 kg	12,699	12,871	172	1.35
Tool + 2 kg	12,821	12,956	135	1.1
Tool + 4 kg	13,122	13,090	32	0.24

### Energy consumption in a manufacturing task

**Figure 12.**
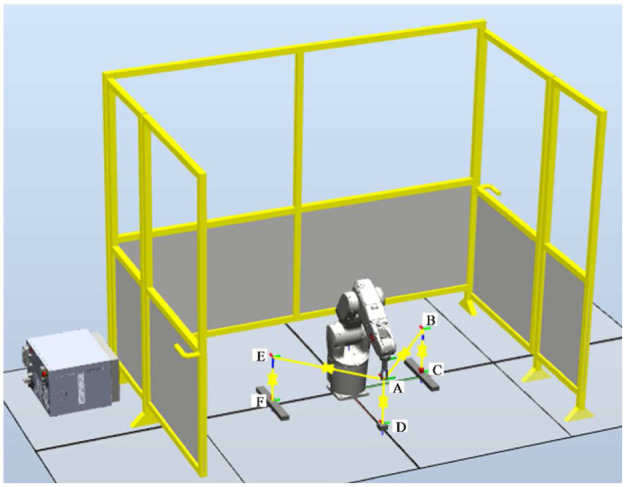
Path of the robot in a manufacturing task.

A test is done to test the impact of robot speed on the energy consumption of the robot in a manufacturing task. The robot is commanded to move according to Figure 12 to fulfill a pick-and-place task. The path of the robot is denoted as follows: (1)A – (2)B – (3)C – (4)B – (5)A – (6)D – (7)A – (8)D – (9)A – (10)E – (11)F – (12)E – (13)A. The robot is installed with a tool. From (3)C, the robot carries a load. After (5)A, the robot releases the load. After (7)A, the robot carries a heavier load. After 10(E), the robot releases the load again.

**Figure 13.**
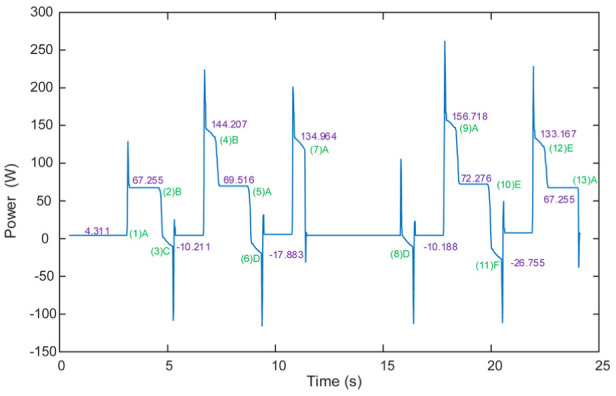
Simulated power of the motors when the speed is 0.5 m/s.

An example of the simulated power of the motors when the speed is 0.5 m/s is shown in Figure 13. The green texts indicate the via points in the robot trajectory and the pink texts indicates the power value (the unit is W) during the movement of the robot. It can be seen from Figure 13 that from (1)A to (2)B the robot carries no load and the simulated power of the motors is 67.255 W. From (12)E to (13)A, the robot carries no load and the simulated power of the motors is approximately equal to that from (1)A to (2)B. From (4)B to (5)A, the robot carries a load and the power is 69.516 W which is larger than that from (1)A to (2)B. From (9)A to (10)E, the robot carries a heavier load and the power is larger than that from (4)B to (5)A. These results show that the simulated power reflects the characteristics of the robot motor power. In the future, we will perform quantitative comparison between the simulation results and the measured ones.

**Figure 14.**
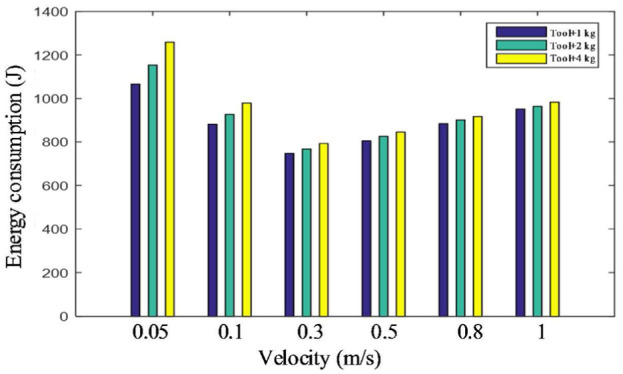
Energy consumption of the motors with different loads and speeds.

Different speeds (0.05, 0.1, 0.3, 0.5, 0.8, and 1 m/s) and loads (0, 2, and 4 kg) are used to perform the task and the energy consumption of each task is shown in Figure 14. It shows that the energy consumption increases with the increase of the load. When the load is the same, the energy consumption of the robot varies with the speed. Too big or too small speed both increase the energy consumption of the robot. This means that the speed should be appropriate to achieve lower energy consumption for the robot. However, the optimal speed for energy consumption reduction may affect the productivity. Therefore, a trade-off is needed in this situation.

## Conclusion

This article has analyzed the energy consumption characteristics of industrial robot. The main focus is the energy consumption modeling of the motor which changes with the trajectory of the robot and can be optimized without causing physical changes to the robot. A method for parameter identification of industrial robot based on power data rather than joint torque is proposed. ABB IRB 1200 is used to demonstrate the method. The simulation results from RobotStudio show the effectiveness of the proposed method. Based on the parameter identification, the energy consumption model of the robot is obtained. The simulated results show that the energy consumption model shows satisfactory accuracy. In the future, we will compare the simulated power with the measured one quantitatively. The relation between the speed of the robot and its energy consumption is analyzed. The calculated results show that the speed should not be too high or too low to achieve optimal energy consumption. However, the productivity should also be considered in this situation. This is a trade-off problem and will be studied in the future research.
